# A new species of the genus *Caissa* Hering, 1931 from Yunnan, China (Lepidoptera, Limacodidae)

**DOI:** 10.3897/zookeys.951.53151

**Published:** 2020-07-22

**Authors:** Jun Wu, Chun-Sheng Wu, Hui-Lin Han

**Affiliations:** 1 School of Forestry, Northeast Forestry University, Harbin, 150040, China Northeast Forestry University Harbin China; 2 Key Laboratory of Zoological Systematics and Evolution, Institute of Zoology, Chinese Academy of Sciences, Beijing 100101, China Institute of Zoology, Chinese Academy of Sciences Beijing China

**Keywords:** *
Caissa
*, China, Lepidoptera, Limacodidae, new species

## Abstract

A new species, *Caissa yunnana***sp. nov.** is described from Yunnan Prov., China. The new species is illustrated with images of the adult habitus and male genitalia, and compared with the similar species *C. caissa* Hering, 1931. A world checklist of the genus *Caissa* Hering, 1931 is provided

## Introduction

The genus *Caissa* Hering, 1931, a member of the family Limacodidae, is based on the type species *C. caissa* Hering, 1931. Hering (1931) first established the genus *Caissa*, which included two newly described species, *C. caissa* and *C. gambita* from India. Later, Yoshimoto (1994) described another species, *C. medialis* from Nepal; Orhant (2000) described *C. parenti* from Myanmar; Solovyev and Witt (2009) described two species, *C. aurea* and *C. bezverkhovi* from Vietnam; Solovyev and Saldaitis (2014) provided several images of adults and genitalia in their paper, including of the type species. The species *C. fasciatum* (Hampson, 1893) was originally described as *Ceratonema fasciatum* by Hampson (1892), then Solovyev (2009) moved it to the genus *Caissa*. Recently, Irungbam et al. (2017) reported the distribution of *C. fasciatum* in Bhutan. In China, the first recorded species of this genus was *C. gambita* (Cai 1981). Subsequently, three new species (*C. longisaccula* Wu & Fang, 2008, *C. caii* Wu & Fang, 2008 and *C. staurognatha* Wu, 2011) and one newly recorded species (*C. parenti* Orhant, 2000) were reported (Wu and Fang 2008; Wu 2011). In 2013, another species, *C. kangdinga* Solovyev & Saldaitis, 2013, was described from Kangding in Sichuan, China.

The features common to the genus are filiform male antennae, labial palpus somewhat appressed and upcurved, not quite reaching the vertex. The forewing has an obvious dark medial band, and the hindwing has a dark tornal spot. The species of the genus are diverse in external morphological characters, in wing venation, and in male genitalia. According to Solovyev and Witt (2009), three species groups can be distinguished within the genus by external characters and male genitalia. The first group contains the type species *C. caissa* and the new species *C. yunnana* sp. nov. This group is characterized by the following characters: forewing ground color is dark brown, with unequal-sized white patches; the tornus contains 2 to 4 whitish spots; the terminal area is white, forming a nearly right triangle; in the male genitalia, the bifurcation of the sacculus process is thin, and the terminal part of the juxta is strongly sclerotized, with dense black spurs; the phallus is slightly curved, and the coecum is very short.

The second group includes *C. fasciatum*, *C. gambita*, *C. longisaccula*, *C. aurea* and *C. bezverkhovi*. This group is characterized as follows: the forewing ground color is pale yellow, with an oblique pale medial line, which is embedded in the black-brown medial band, running from 1/2 costal margin to 1/3 inner margin; the postmedial line is very indistinct or almost absent; there are 1 or 2 small black spots near the apex of the terminal line and a dark spot near the base of vein CuP; in the male genitalia, the cucullus is round; the sacculus is significantly inflated, about half the length of the valva; the broad bifurcation of the sacculus process is triangular blade shaped or finger-like; the phallus is curved, and the coecum is short.

The third group includes *C. medialis*, *C. parenti*, *C. caii*, *C. staurognatha* and *C. kangdinga*. This group is characterized as follows: the forewing medial band is black or brown, without an embedded pale medial line; the inner border of the medial band runs from 1/3 the distance from the wing base on the costal margin, to 2/5 from the wing base on the inner margin, and the outer border runs from 2/5 from the wing base on the costal margin, to 3/5 from the wing base on the inner margin; the arcuate postmedial line is distinct; in the male genitalia, the gnathos is varied, well developed or reduced; the terminal part of the juxta has a distinct elongation; the inflated part of the sacculus is short, about 1/3 length of the valva; the sacculus process is not forked; the phallus is straight, and the coecum is rather longer than in the other two groups, over 1/3 length of the phallus.

Here we describe a new species from Yunnan Province in southwest China. Based on the features of the forewing with unequal-sized white patches, the white terminal area forming a nearly right triangle, and the terminal part of juxta strongly sclerotized, U-like, with dense black spurs, it is assigned to the first group. The genus now includes 11 species, six of which are distributed in China.

## Material and methods

The specimens were collected at 220 V/450 W mercury light and DC black light in Yunnan Province, China. Standard methods for dissection and preparing of the genitalia slides were used (described by Kononenko and Han 2007). Specimens were photographed using a Nikon D700 camera; the genitalia slides were photographed using an Olympus photo microscope aided by the Helicon Focus software, and further processed in Adobe Photoshop CS6. The type material of the new species is deposited in the collection of Northeast Forestry University, Harbin, China (NEFU).

## Taxonomic account

### Genus Caissa

Taxon classificationAnimaliaLepidopteraLimacodidae

Hering, 1931

7C98B023-81DB-5975-A30A-E1D1BAB093E7

Caissa Hering, 1931, in Seitz, Macrolep. World, 10: 670, 700. Type species: Caissa caissa Hering, 1931 [India: Khasis Hills].

### Caissa yunnana sp. nov.

Taxon classificationAnimaliaLepidopteraLimacodidae

357299E0-CA01-5DB2-A615-160E7E0A6742

http://zoobank.org/2BE08BA2-B4B7-4711-B2F0-D6BF26899AF5

Figures 1 , 3

#### Holotype.

♂, China, Yunnan Province, Lvchun County, Mt. Huanglian, 27–31.VII.2018, leg. HL. Han, J. Wu, MR. Li [NEFU], genit. prep. WuJ-069-1.

#### Paratypes.

3♂, same data as holotype [NEFU], genit. prep. for one dissected paratype WuJ-068-1.

#### Description.

**Figures 1–4.**
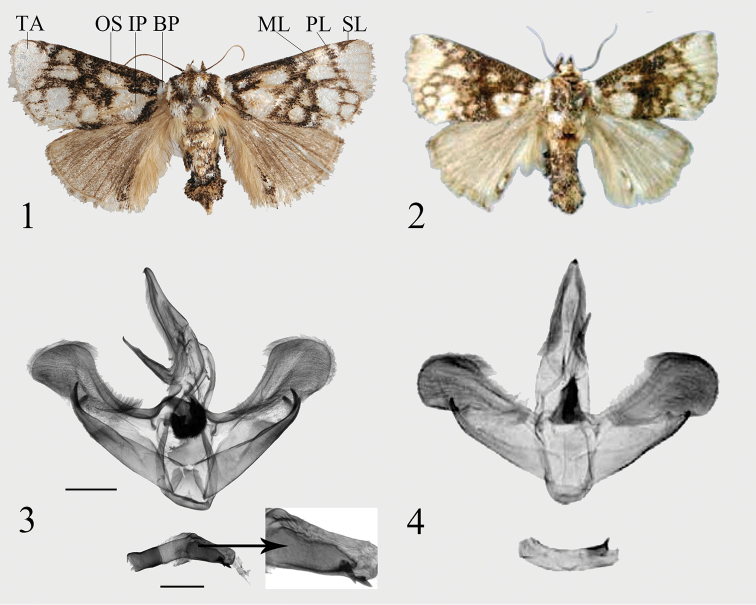
*Caissa* spp., adults: **1***C. yunnana***sp. nov.**, male, holotype **2***C. caissa* Hering, 1931, male, holotype (after Solovyev and Saldaitis 2014); male genitalia: **3***C. yunnana***sp. nov.**, holotype **4***C. caissa* Hering, 1931, holotype (after Solovyev and Saldaitis 2014). Scale bars: 1 mm. (BP: basal patch; IP: inner patch; ML: medial line; OS: orbicular spot; PL: postmedial line; SL: subterminal line; TA: terminal area).

Adult (Fig. 1). Wingspan 29–31 mm in male. Head white, labial palpus and patagium brown. The male antennae filiform, brown; the thorax mixed white and brown, the tegula white with brown margin; dorsally abdomen yellowish brown to dark brown, ventrally white or yellowish brown. Forewing ground color dark brown, with unequal-sized white patches; light-colored basal patch small, not connected with inner patch; orbicular spot large, broadly connected to the larger inner patch; two medium-sized white patches lie in the middle of the forewing, near the inner margin, distal to the medial line which runs from the costal margin at 1/2 the distance from the wing base, to the inner margin at 1/3 from the wing base; arcuate postmedial line from the costal margin at 2/3 from the wing base to the tornus; postmedial area with 4 large whitish spots, the tornus with 2 whitish spots; subterminal line curved strongly inward, from apex to the outer margin at 3/5 from the apex, combined with postmedial line on R_5_; terminal area white, forming a nearly right triangle. Hindwing brown and mixed with a small amount of red, anal area light brown with oblong dark blotch with a white spot inside. The white spot is much smaller than the encircling brown.

**Male genitalia** (Fig. 3). Uncus slender, with a small subapical spur. Gnathos well developed, very straight rod-shaped terminal part with slightly curved apex. The base of valva wider than middle; the cucullus wide and round; costa simple, slightly shorter than valve; sacculus sclerotized and shorter than costa, sacculus process bifurcated, distinctly incurved and hook-shaped. Juxta asymmetrical, the right side of terminal part strongly sclerotized, U-like, with dense black spurs. Saccus inconspicuous. Phallus curved, weakly sclerotized, bent into an obtuse angle in the middle; 1/2 of the terminal part with a strongly sclerotized wedge-shaped area; the terminal part of carina sclerotized, short, cone-shaped; vesica with big cyst, surface covered with small spines, with a big, sclerotized cornutus.

**Female genitalia.** Unknown.

#### Diagnosis.

The new species is similar in appearance to *C. caissa* (Fig. 2), but can be distinguished from the latter by the characters of the forewing and male genitalia, as follows. In *C. yunnana* the light-colored basal patch is small, not connected with the inner patch; orbicular spot large, connected with the inner patch (Fig. 1); in *C. caissa*, the basal patch is bigger than in *C. yunnana*, and it is broadly connected with the inner patch, but not connected with the small orbicular spot. Also, the tornus of *C. yunnana* contains only 2 whitish spots, but the same location in *C. caissa* contains 4 whitish spots. The color of the hindwing in *C. yunnana* is darker than in *C. caissa*.

In the male genitalia, the new species clearly differs from *C. caissa* (Fig. 4) by the sclerotized area of the sacculus being wider than the same area in *C. caissa*; in *C. yunnana* the sacculus process is bifurcate, distinctly incurved and hook-shaped, whereas in *C. caissa* it is short, and although bifurcate, barely hooked. The phallus of *C. yunnana* differs from that of *C. caissa* by the middle being bent into an obtuse angle, 1/2 of the terminal part with a strongly sclerotized wedge-shaped area, and the vesica with a big cyst and a big sclerotized cornutus. The phallus of *C. caissa* is smoothly curved, and the vesica is membranous, without a cyst or cornutus.

#### Distribution.

**Figures 5, 6.**
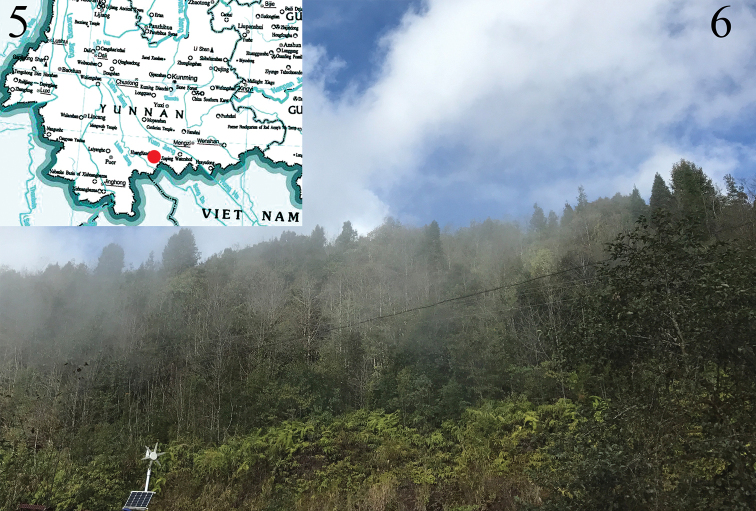
Map and habitat of *C. yunnana* sp. nov. **5** Collecting site of *C. yunnana* sp. nov.: Yunnan Prov., Mt. Huanglian (red dot) **6** Collecting site close to a broad-leaved forest with ferns and shrubs.

China (Yunnan Province: Mt. Huanglian) (Fig. 5).

#### Etymology.

The species is named for its type locality in Yunnan Province, China.

#### Bionomics.

The moths fly in July. The specimens were collected with a light trap close to a broad-leaved forest with ferns and shrubs (Fig. 6).

## World checklist of the genus *Caissa*, with type localities

### Group 1:

*C. caissa* Hering, 1931 (India: Khasis Hills)

*C. yunnana* Wu, Wu & Han, sp. nov. (China: Yunnan)

### Group 2:

*C. fasciatum* (Hampson, 1893) (India: Nágas)

*C. longisaccula* Wu & Fang, 2008 (China: Fujian)

*C. gambita* Hering, 1931 (India: Travancore)

*C. aurea* Solovyev & Witt, 2009 (Vietnam: Lao Cai)

*C. bezverkhovi* Solovyev & Witt, 2009 (Vietnam: Nghe An)

### Group 3:

*C. kangdinga* Solovyev & Saldaitis, 2013 (China: Sichuan)

*C. caii* Wu & Fang, 2008 (China: Shaanxi)

*C. parenti* Orhant, 2000 (Myanmar: Maymyo)

*C. medialis* Yoshimoto, 1994 (Nepal: Kathmandu)

*C. staurognatha* Wu, 2011 (China: Sichuan)

## Supplementary Material

XML Treatment for Caissa

XML Treatment for Caissa yunnana
